# Supplementary material for: Inter-decadal climate variability induces differential ice response along Pacific-facing West Antarctica
Source: Nat Commun. 2023 Jan 16;14:93. doi: 10.1038/s41467-022-35471-3 (PMC9842681; doi:10.1038/s41467-022-35471-3)
Supplement: Supplementary file 1 — Supplementary Information [file 41467_2022_35471_MOESM1_ESM.pdf]

SUPPLEMENTARY INFORMATION

FOR

--

Inter-decadal climate variability induces differential ice response along Pacific-facing West Antarctica

--

Frazer D.W. Christie[1,2], Eric J. Steig[3], Noel Gourmelen[2], Simon F. B. Tett[2] and Robert G. Bingham[2]

[1]Scott Polar Research Institute, University of Cambridge, Cambridge, UK, CB2 1ER

[2]School of GeoSciences, University of Edinburgh, Edinburgh, UK, EH8 9XP

[3]Department of Earth & Space Sciences, University of Washington, Seattle, WA, USA, 98195-1310

Corresponding author: Frazer D. W. Christie (fc475@cam.ac.uk)

Contents:

Supplementary Figs. 1-9

Supplementary Table 1

SUPPLEMENTARY FIGURES:

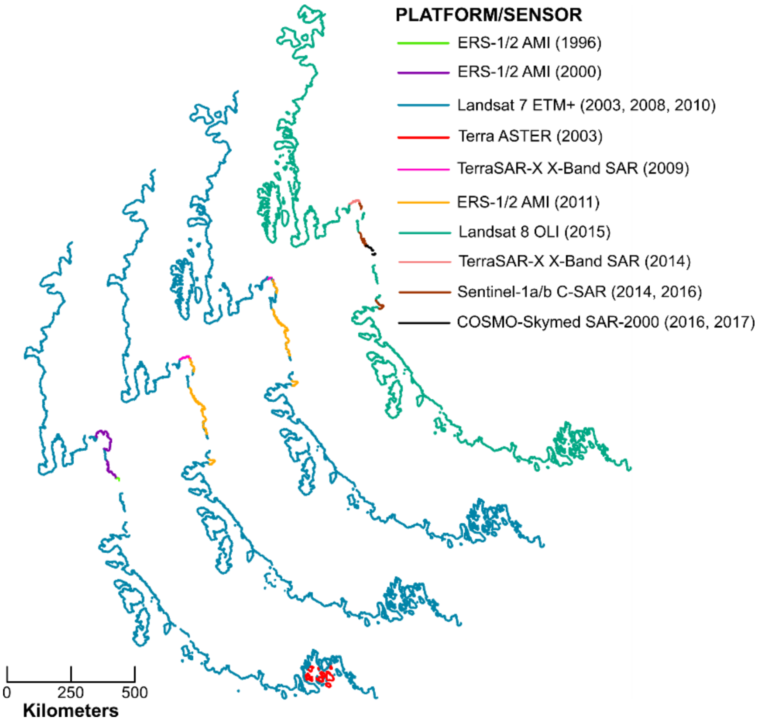

Supplementary Fig. 1 | Grounding-line location observations used in this study. Figure shows the spatial coverage of the various satellite-derived datasets used to calculate rates of grounding-line migration (see Methods for further information).

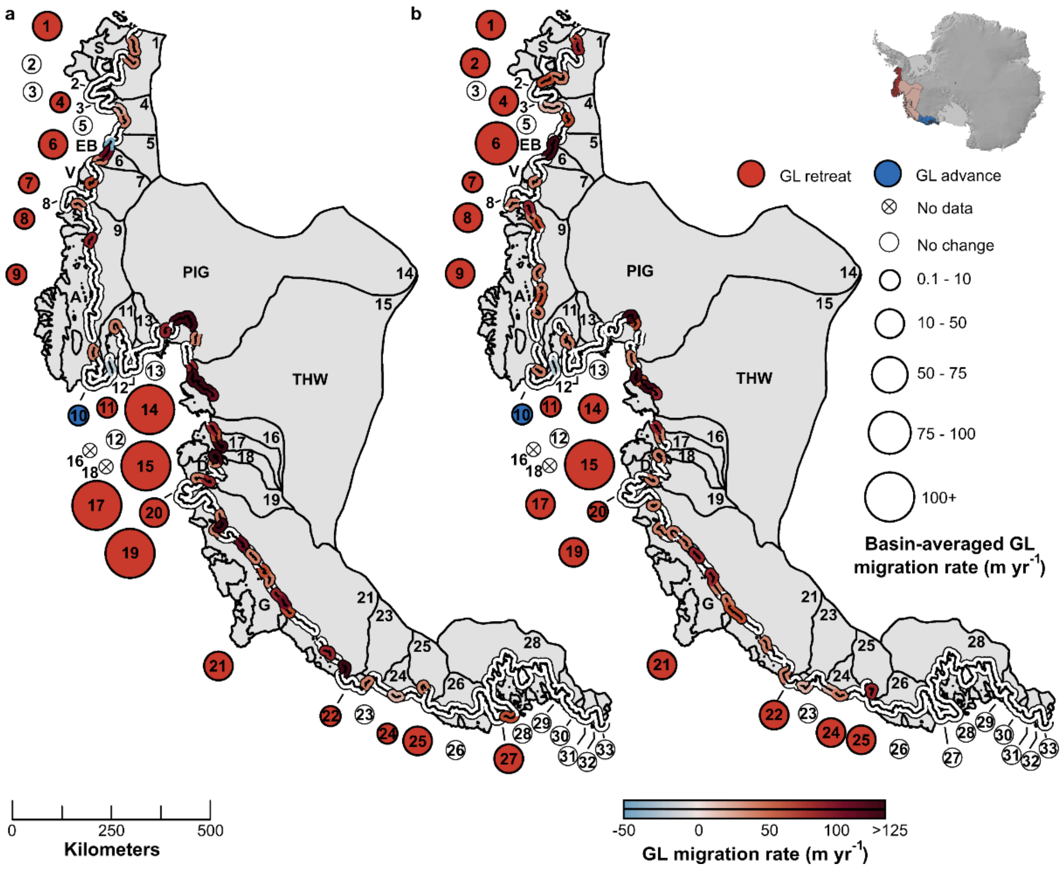

Supplementary Fig. 2 | Grounding-line migration along West Antarctica's Pacific-facing margin, 2003-2015. Same as Fig. 1, but showing net grounding-line (GL) migration rates (m yr^{-1}) per 30 km segment along the coast for the periods ***a***, 2003-2008 and ***b***, 2010-2015. Red denotes grounding-line retreat; blue, advance; white, negligible change within satellite error bounds (Methods, see also Supplementary Fig. 3 and Supplementary Data 2). Basin-averaged rates of grounding-line migration (numbered circles) are also shown. Note the non-linear scale used to emphasise slowdown (speedup) in the Amundsen (Bellingshausen) Sector. Note that the glacial basins shown are from MEaSUREs[83] but for ease of reference we have numbered them 1-33 from east to west.

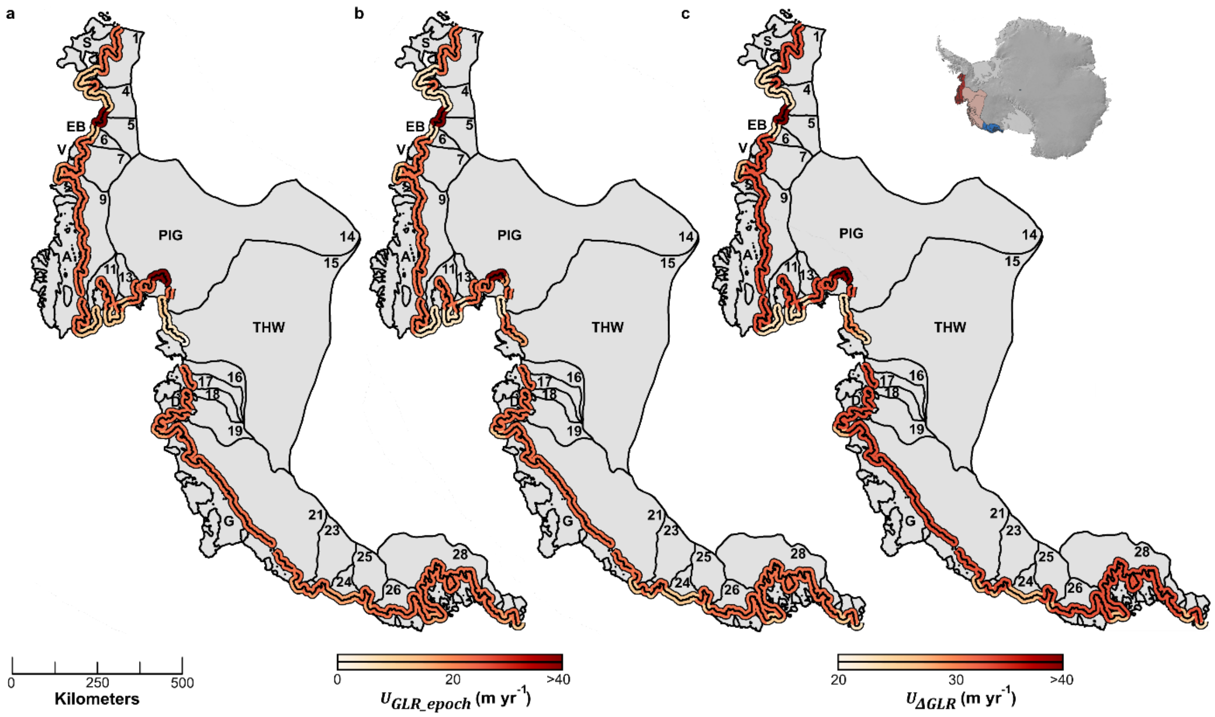

Supplementary Fig. 3 | Grounding-line migration rate uncertainty. Per-segment propagated uncertainty values, U_{GLR_epoch}, associated with our grounding-line migration rate observations spanning ***a***, 2003-2008 and ***b***, 2010-2015, respectively (cf. Supplementary Fig. 2). ***c***, same as *a*-*b*, but showing between-epoch propagated uncertainty, $U_{\Delta GLR}$, associated with our grounding-line migration rate change observations presented in Fig. 1 (see Methods and Supplementary Data 2 for further information). Select basin number and site labels are shown for ease of comparison with Fig. 1 and Supplementary Fig. 2.

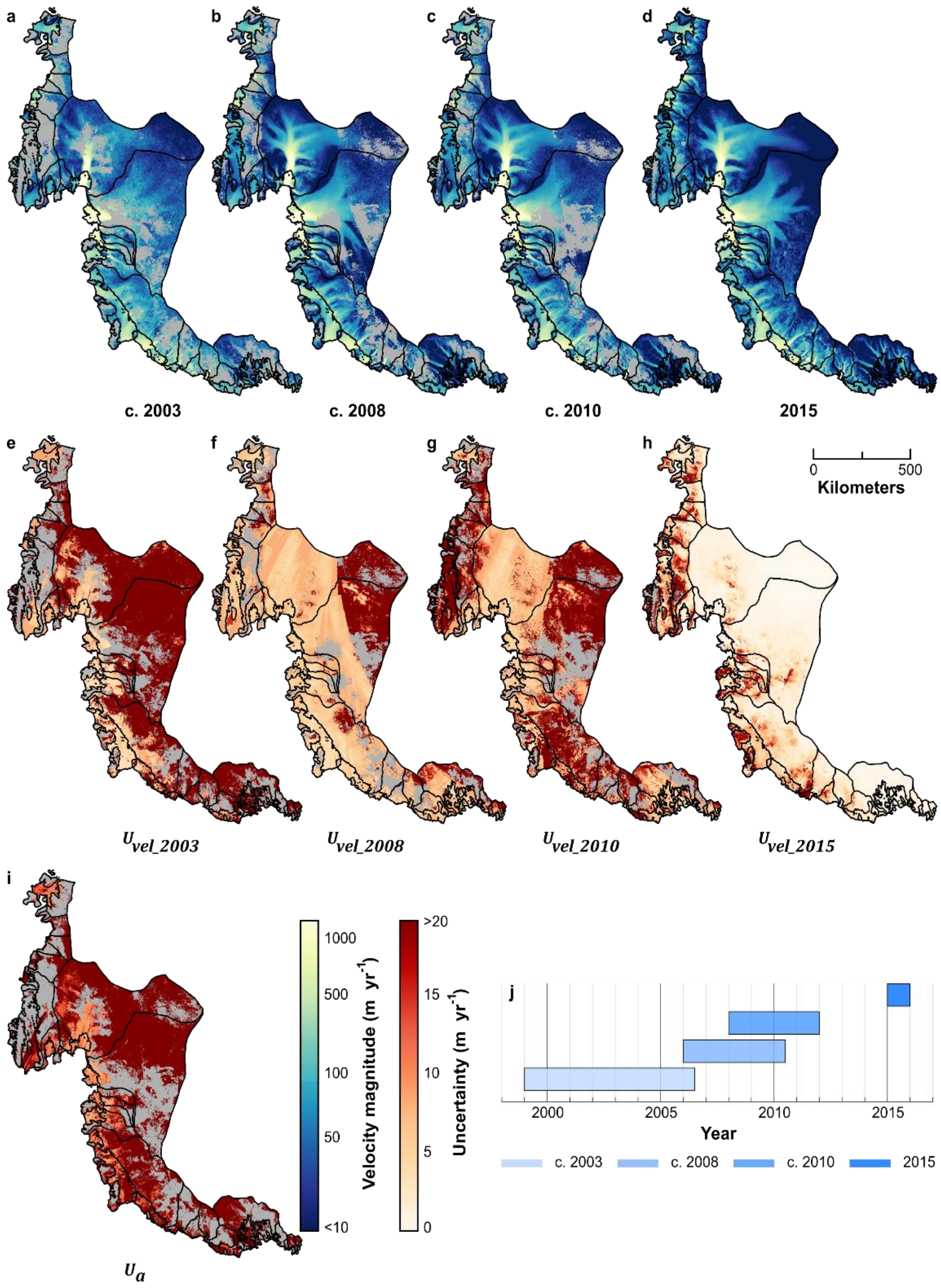

Supplementary Fig. 4 | Velocity data used in this study. ***a-d***, stacked velocity magnitude grids over Pacific West Antarctica used to produce the ice-flow acceleration map shown in Fig. 1. ***e-h***, corresponding rates of uncertainty (U_{vel}) associated with panels *a-d* and, ***i***, that of Fig. 1 (U_a). Uncertainties are sensitive to the number of input observations associated with data stacking and so should be considered a relative measure of data quality rather than absolute error (see Methods and Supplementary Table 1 for further information). ***j***, timeline showing temporal coverage of the velocity mosaics used to generate the grids shown in *a-i*.

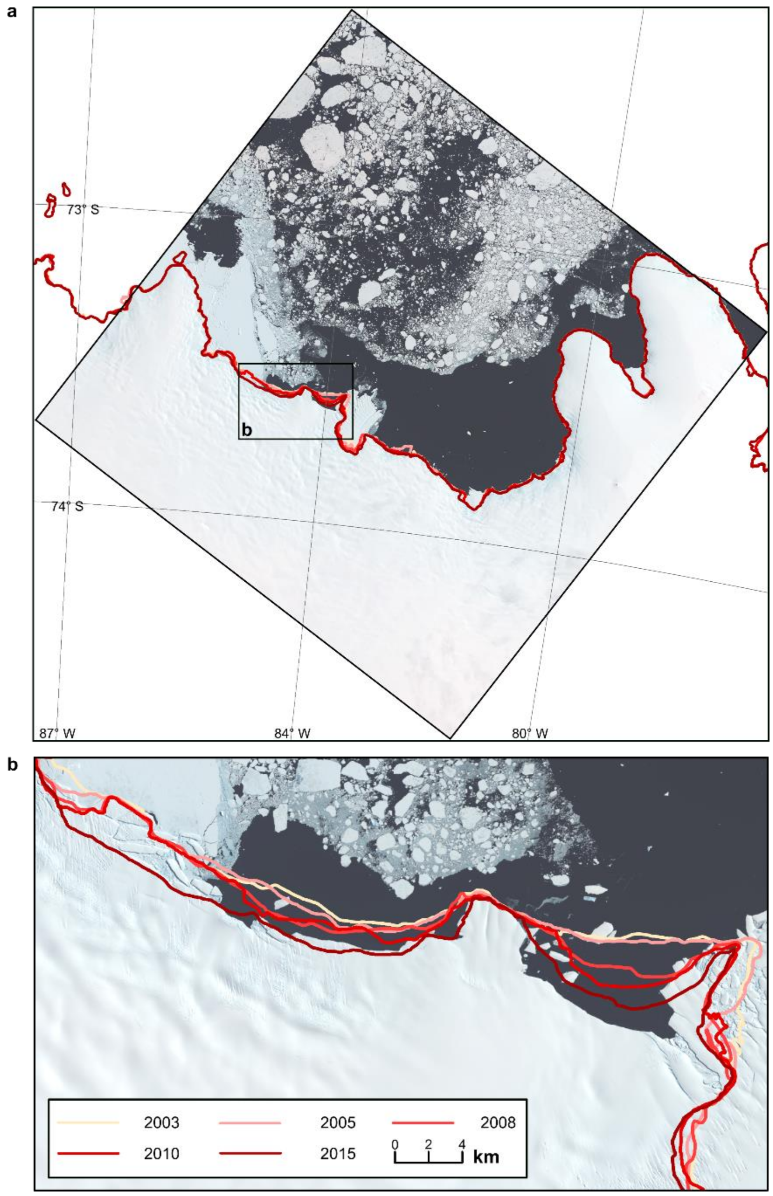

Supplementary Fig. 5 | Grounding-line retreat in the Eltanin Bay region of the Bellingshausen Sector. ***a***, Landsat-derived I_b change along an 'ice-wall'-type grounding line[49]. ***b***, detail showing magnitude of grounding-line retreat over the observational period. Panels *a* and *b* are superimposed over a Landsat 8 image dated 26th December 2019, revealing the continued retreat of this region since 2015.

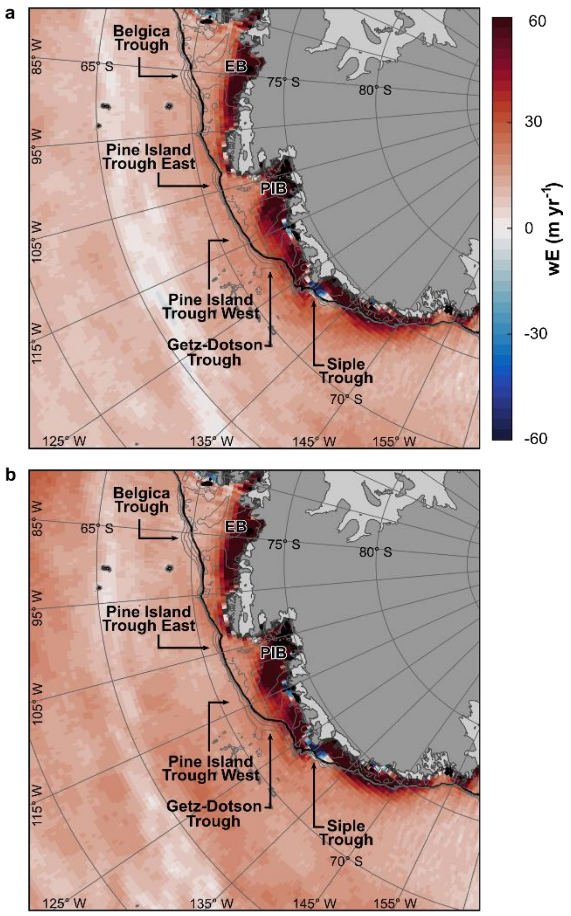

Supplementary Fig. 6 | Ekman vertical velocity along Pacific-facing West Antarctica, 2003-2015. Mean ERA5-derived Ekman vertical velocity, wE (m yr^{-1}; negative denotes downwelling), over the periods ***a***, 2003-2008 and ***b***, 2010-2015. Contours and site labels same as Fig. 2c. Dark grey denotes no data.

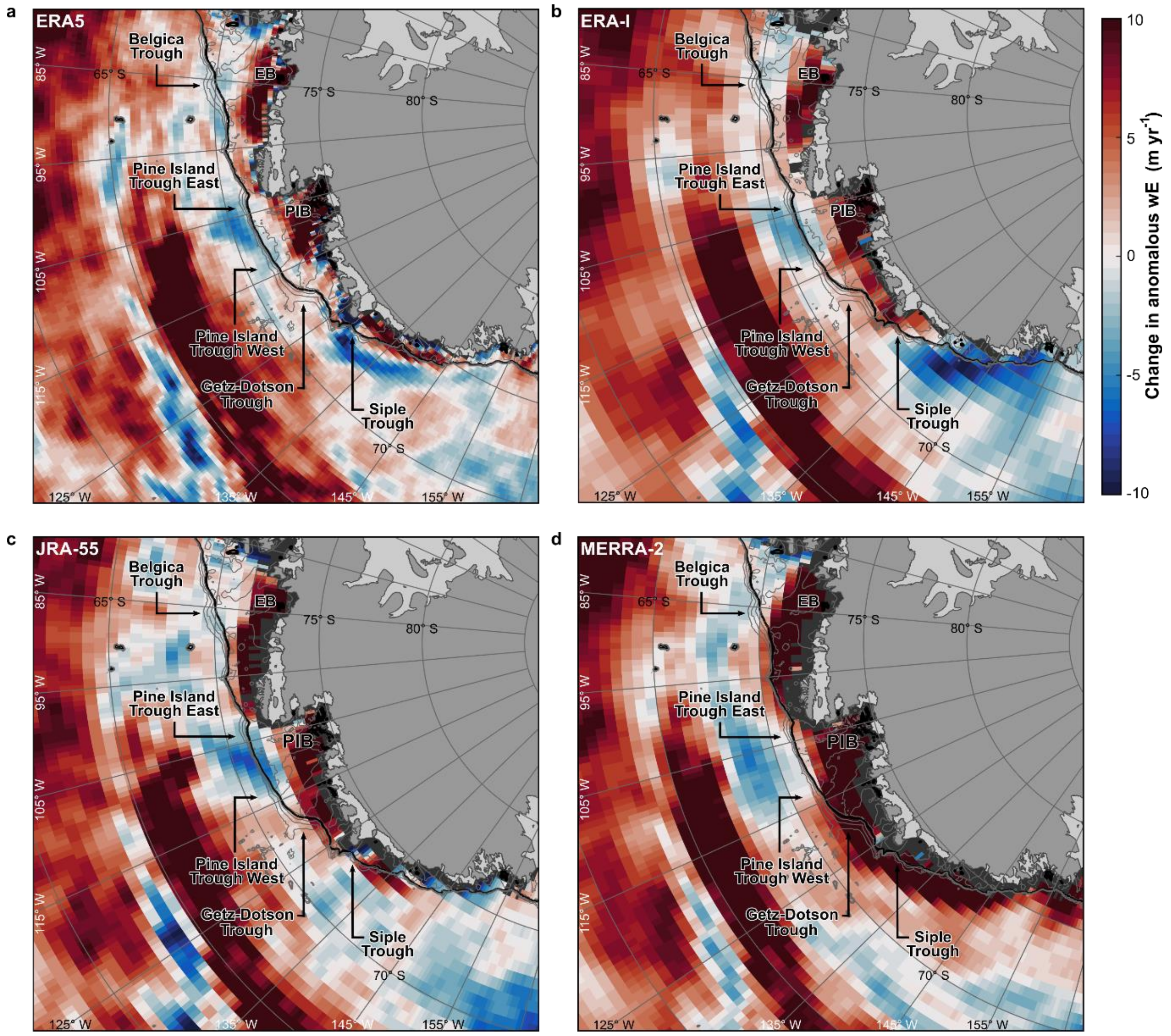

Supplementary Fig. 7 | Interdecadal change in anomalous Ekman vertical velocity along Pacific-facing West Antarctica, 2003-2015. ***a***, Same as Fig. 2c but shown at native model resolution. ***b-d***, Same as *a*, but derived from the *b*, ERA-Interim, *c*, JRA-55 and *d*, MERRA-2 reanalyses. Negative wE denotes less upwelling during the period 2010-2015 compared to 2003-2008; dark grey, no data.

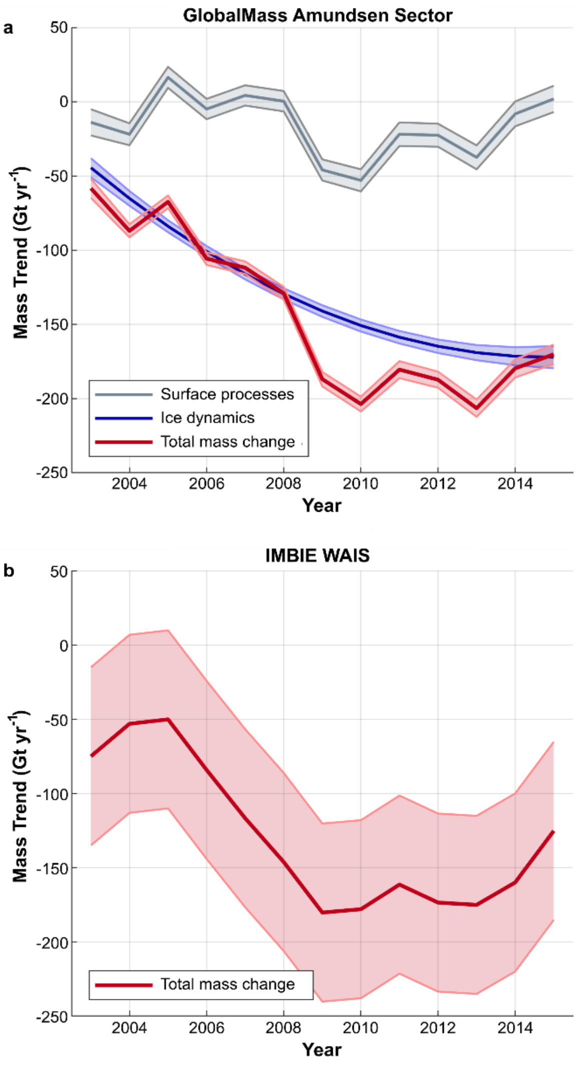

Supplementary Fig. 8 | West Antarctic mass trends, 2003-2015. Figure shows total mass-balance trend estimates (red) for ***a***, the Amundsen Sector (drainage basins 320, 321 and 322 in ref. 55) as resolved from Bayesian hierarchical modelling (after ref. 54; Methods). Contributions from surface processes (grey) and ice dynamics (blue) are also shown. For reference, IMBIE 2016 mass-balance trend estimates integrated over the entire West Antarctic Ice Sheet[1] are also shown (***b;*** Methods). In both plots, shading denotes 1σ. Note the close agreement between both plots, whose trends reflect the reduced acceleration of the Amundsen Sector's glaciers since *c*. 2010 (cf. Fig. 1).

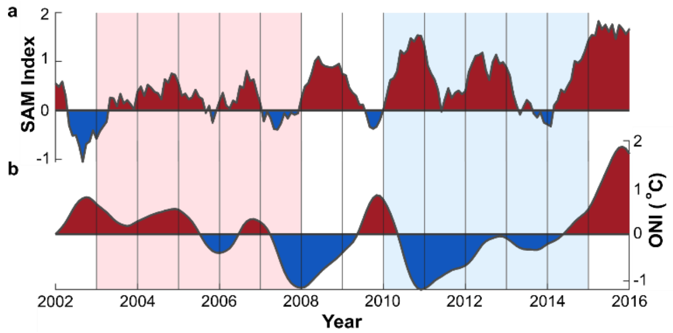

Supplementary Fig. 9 | Southern Annular Mode and El Niño-Southern Oscillation forcing. Figure shows changes (12-month running means) in the polarity of ***a***, the Southern Annular Mode (SAM) and ***b***, the El Niño-Southern Oscillation (ENSO) between 2003 and 2015 (Methods). Positive ENSO denotes El Niño-like conditions; negative, La Niña. Translucent red and blue patches delimit the periods 2003-2008 and 2010-2015, respectively, which correspond to the timing of our glaciological observations. Note the close synchronicity between our observations of reduced grounding-line retreat and ice-flow acceleration rates in the Amundsen Sector between 2010-2015 (Fig. 1) and the transition towards strong SAM+ and La Niña-like conditions *c*.2010 (see main text for further discussion).

Supplementary Table 1 | Velocity mosaics used in this study.

Dataset	Dataset year(s)	Measurement type	Native Resolution (m)	Source	Velocity stack year
MEaSUREs InSAR-Based Ice Velocity of the Amundsen Sea Embayment, Antarctica, Version 1	2000, 2002	Speckle tracking	450	Rignot et al. (51)	*c*. 2003
MEaSUREs Annual Antarctic Ice Velocity Maps, 2005-2017, Version 1	2005-2006	Speckle tracking/InSAR	1000	Mouginot et al. (52)	*c*. 2003
NASA ITS_LIVE Annual Antarctic Ice Velocity Map	1999, 2000, 2001, 2002, 2003, 2004, 2005	Landsat 7-based feature tracking	240	Gardner et al. (8, 53)	*c*. 2003
MEaSUREs InSAR-Based Ice Velocity of the Amundsen Sea Embayment, Antarctica, Version 1	2006-2007, 2007-2008, 2008-2009, 2009-2010	Speckle tracking	1000	Rignot et al. (51)	*c*. 2008
MEaSUREs Annual Antarctic Ice Velocity Maps, 2005-2017, Version 1	2007-2008, 2008-2009, 2009-2010	Speckle tracking/InSAR	450	Mouginot et al. (52)	*c*. 2008
NASA ITS_LIVE Annual Antarctic Ice Velocity Map	2007, 2008, 2009	Landsat 7-based feature tracking	240	Gardner et al. (8, 53)	*c*. 2008
MEaSUREs InSAR-Based Ice Velocity of the Amundsen Sea Embayment, Antarctica, Version 1	2008-2009, 2009-2010, 2010-2011, 2011-2012	Speckle tracking	1000	Rignot et al. (51)	*c*. 2010
MEaSUREs Annual Antarctic Ice Velocity Maps, 2005-2017, Version 1	2009-2010, 2010-2011, 2012-2013	Speckle tracking/InSAR	450	Mouginot et al. (52)	*c*. 2010
NASA ITS_LIVE Annual Antarctic Ice Velocity Map	2008, 2008, 2010, 2011, 2012	Landsat 7-based feature tracking	240	Gardner et al. (8, 53)	*c*. 2010
NASA ITS_LIVE Annual Antarctic Ice Velocity Map	2015	Landsat 8-based feature tracking	240	Gardner et al. (8, 53)	2015

InSAR denotes Interferometric Synthetic Aperture Radar-based imaging techniques
