# Supplementary material for: Inter-decadal climate variability induces differential ice response along Pacific-facing West Antarctica
Source: Nat Commun. 2023 Jan 16;14:93. doi: 10.1038/s41467-022-35471-3 (PMC9842681; doi:10.1038/s41467-022-35471-3)
Supplement: Supplementary file 3 — Description of Additional Supplementary Files [file 41467_2022_35471_MOESM3_ESM.pdf]

Description of Additional Supplementary Files

File Name: Supplementary Data 1

Description: Landsat and TerraSAR-X data used in this study

File Name: Supplementary Data 2

Description: Pacific WAIS grounding-line migration values.
